# Successful Treatment of Vesico-Vaginal Fistula by Cauterization

**Published:** 1858-01

**Authors:** 


*Successful Treatment of Vesico-Vaginal Fistula by Cauterization.*—We notice in the Dec. number of the Cincinnati Medical Observer, a report of a case, by Dr. G. Bruhl, of vesico-vaginal fistula, successfully treated by cauterization with the nitrate of silver. Dr. Bruhl saw the patient fourteen days after delivery, who suffered under a transverse fistula about 1¼ inches long, a little above the centre of the vagina. As the patient was unwilling to submit to an operation, she was treated by the application of solid nitrate of silver, twice a week. In four weeks the opening had completely closed. F.

